# Evaluation of the effect of perioperative nursing intervention for esophageal cancer patients based on the ADOPT nursing model

**DOI:** 10.1097/MD.0000000000048024

**Published:** 2026-03-27

**Authors:** Pazilaiti Saideng, Yuan Lv, Gulizhaernuer Aierken, Mayire Tuersun, Minawaer Wujiaji

**Affiliations:** a Cardiovascular Surgery Center ICU, The First People’s Hospital of Kashi Prefecture, Kashi, Xinjiang, China; b Nursing Department, The First People’s Hospital of Kashi Prefecture, Kashi, Xinjiang, China; c Thoracic Surgery Department, The First People’s Hospital of Kashi Prefecture, Kashi, Xinjiang, China.

**Keywords:** ADOPT model of care, esophageal cancer, perioperative care, self-care competence, self-efficacy, symptom management

## Abstract

This study evaluated the effectiveness of the Attitude, Definition, Open mind, Planning, Try it out (ADOPT) nursing model during the perioperative period in patients with esophageal cancer, focusing on self-management efficacy, self-care ability, and symptom clusters. Seventy-six patients undergoing radical resection between November 2024 and September 2025 were assigned to either the ADOPT group or the conventional care group (38 patients each) according different. Both groups received routine perioperative nursing, while the ADOPT group additionally implemented systematic interventions based on the 5 components of ADOPT. Patient outcomes were assessed using the Strategies Used by People to Promote Health, Exercise of Self-Care Agency, and MD Anderson Symptom Inventory scales on day 2 of admission, postoperative day 1, and prior to discharge. Baseline characteristics and surgical parameters were comparable between groups. At discharge, patients in the ADOPT group demonstrated significantly greater improvements in self-management efficacy and self-care ability, as well as reduced symptom severity and functional limitations, compared with the conventional group (*P* < .05). These findings indicate that the ADOPT nursing model effectively enhances patient self-management and self-care capacity while alleviating symptom burden, providing a systematic and individualized framework to optimize perioperative care for esophageal cancer.

## 1. Introduction

Esophageal cancer is a major gastrointestinal malignancy with a particularly high burden in East Asia. GLOBOCAN 2020 data reported over 600,000 new cases and 544,000 deaths worldwide, ranking it among the most prevalent and lethal cancers globally.^[1]^ China accounts for nearly half of the global cases, with a 5-year survival rate still below 30%.^[2]^ Surgical resection remains the primary curative option; however, patients frequently encounter multiple postoperative challenges, such as malnutrition, dysphagia, impaired respiratory function, cancer-related fatigue, pain, and psychological distress.^[3]^ These complications not only delay recovery but also substantially diminish quality of life. Therefore, optimizing perioperative nursing interventions to alleviate symptoms and enhance recovery has become a critical focus in clinical care.

In recent years, it has become evident that symptoms in esophageal cancer patients often occur as interrelated “symptom clusters,” including pain, insomnia, anxiety, and fatigue, which frequently coexist and exert synergistic adverse effects.^[4]^ Prospective studies have demonstrated that these clusters are strong predictors of quality of life and functional recovery.^[5,6]^ Consequently, traditional nursing approaches focused on isolated symptom management are insufficient to address patients complex needs. A shift toward systematic, continuous, patient-centered, and integrated nursing models is therefore urgently required.

Multimodal rehabilitation and systematic interventions have demonstrated clear benefits in nursing practice. Chen et al^[7]^ reported that a nurse-led multimodal rehabilitation program significantly improved quality of life, sleep, and psychological outcomes in patients receiving postoperative radiotherapy. Similarly, a randomized controlled trial showed that such interventions alleviated anxiety and depression while enhancing functional recovery.^[8]^ Nevertheless, most existing studies emphasize single components or short-term outcomes, and a comprehensive framework for whole-process, closed-loop care remains lacking.

The Attitude, Definition, Open mind, Planning, Try it out (ADOPT) model is a structured nursing framework that creates a closed-loop process through 5 stages: assessment, decision, optimization, planning, and evaluation. It emphasizes individualized care and multidisciplinary collaboration, and has been increasingly applied in chronic disease management and certain oncology settings.^[9,10]^ Evidence indicates that the ADOPT model can improve patients self-efficacy, treatment adherence, and rehabilitation outcomes.^[11]^ However, its application in the perioperative care of esophageal cancer patients remains largely unexplored.

Quantitative assessment tools are widely applied in nursing intervention research. The Strategies Used by People to Promote Health (SUPPH) scale has been validated for evaluating self-management efficacy in cancer patients.^[12]^ The Exercise of Self-Care Agency (ESCA) scale assesses self-care ability and is extensively used in oncology nursing studies.^[13]^ The MD Anderson Symptom Inventory (MDASI) provides a comprehensive evaluation of symptom severity and functional impairment, making it a key instrument in symptom cluster research.^[14,15]^ Integrating these tools enables a multidimensional assessment of patients self-management, self-care capacity, and symptom control.

Beyond the use of standardized tools, recent studies highlight the importance of nursing interventions in enhancing self-efficacy and self-management. Cui et al^[6]^ reported that both physical and psychological symptoms are strongly associated with cancer-related fatigue, indicating that interventions should address multidimensional needs. Lawler et al^[16]^ found that self-efficacy is critical for sustaining self-management behaviors in cancer patients during extraordinary situations such as the COVID-19 pandemic. Similarly, Zhou et al^[17]^ demonstrated that a comprehensive self-efficacy–oriented intervention in gastric cancer patients improved self-management capacity and quality of life. Moreover, digital health strategies, such as instant messaging–based self-management programs, have shown benefits in improving self-efficacy and quality of life.^[18]^ Collectively, these findings underscore the replicable value of systematic, self-efficacy–centered interventions in cancer care.

Nevertheless, existing research on perioperative nursing for esophageal cancer patients shows notable limitations: most interventions target isolated symptoms or single recovery stages, without full-cycle continuity; the evidence is largely derived from empirical studies or small cohorts, with a paucity of randomized controlled trials grounded in systematic frameworks; and outcome measures remain narrow, rarely integrating self-efficacy, self-care ability, and symptom clusters into comprehensive evaluations. Accordingly, there is an urgent need for a scientifically grounded, actionable nursing model capable of addressing patients multidimensional health needs through holistic intervention.

Building on conventional nursing care, this study incorporated the ADOPT model to establish a systematic intervention pathway encompassing the entire perioperative process – from preoperative preparation to intra- and postoperative management. Patients self-management efficacy, self-care ability, and symptom cluster changes were evaluated using 3 validated instruments (SUPPH, ESCA, and MDASI) at admission (day 2), the 1st postoperative day, and before discharge. The main innovations of this study are as follows: the first application of the ADOPT model to perioperative nursing for esophageal cancer in China; the use of a randomized controlled design to provide evidence-based validation of the model effectiveness; and the integration of multi-scale assessments to comprehensively evaluate the impact of nursing interventions on recovery quality. This approach is expected to establish a replicable and generalizable nursing pathway for esophageal cancer patients, ultimately improving postoperative recovery and long-term survival outcomes.

## 2. Methodology

### 2.1. Study design and participants

This was a single-center conducted at our hospital, enrolling patients with esophageal cancer who underwent radical resection between November 2024 and September 2025.

Inclusion criteria: Pathologically confirmed primary esophageal cancer with radical resection; age ≥ 18 years; complete clinical data available; conscious and able to cooperate with nursing care and study procedures; expected survival ≥ 3 months; provision of written informed consent.

Exclusion criteria: Presence of other malignancies or severe systemic disease; previous radiotherapy or immediate need for postoperative radiotherapy; severe psychiatric disorders or cognitive impairment; participation in other interventional clinical trials.

Sample size calculation: Sample size was estimated using the formula for comparing means between 2 independent groups. With α = 0.05 (2-sided), statistical power = 0.80, and an effect size of 0.65 derived from a preliminary pilot study, the required number was 34 participants per group. Allowing for a 10% dropout rate, the final target enrollment was 38 patients per group, for a total of 76.

Due to the nature of the nursing intervention, blinding of patients and nursing staff was not feasible. However, statisticians responsible for data analysis remained blinded to group allocation to minimize assessment bias.

### 2.2. Nursing model

Conventional nursing group: Patients received routine perioperative care, including preoperative health education, postoperative positioning, pain management, vital sign monitoring, complication prevention, and discharge guidance.

ADOPT nursing group: On the basis of conventional care, the ADOPT model was implemented, following 5 sequential components – Attitude, Definition, Open mind, Planning, and Try it out – emphasizing patient-centered, systematic, and individualized nursing interventions. Specific measures included:

1. Attitude: Nurses fostered patient confidence in treatment and recovery through positive communication and humanistic care. Before surgery, the responsible nurse conducted face-to-face discussions with patients and families to assess psychological status and individual needs, helping them establish a positive outlook and reduce anxiety.
2. Definition: Nursing priorities and risk factors were clarified according to patients condition, surgical approach, and comorbidities, with explicit goals set. For example, in elderly patients or those with chronic diseases, emphasis was placed on cardiopulmonary monitoring and complication prevention.
3. Open mind: Care emphasized individualization and multidisciplinary collaboration, encouraging patients to express their needs and preferences. Preoperative seminars integrated input from surgeons, anesthesiologists, and nutritionists, with nursing measures flexibly adapted to patient wishes rather than rigidly applying a single protocol.
4. Planning: Based on nursing goals and patient-specific conditions, detailed perioperative care plans were developed. These included preoperative psychological support and functional exercise, intraoperative cooperation and positioning, postoperative analgesia and mobilization, nutritional support, drainage management, and early complication warning. Plans were explained to patients and families preoperatively to improve compliance.
5. Try it out: Care plans were implemented stepwise during the perioperative period. Examples included multimodal analgesia for pain relief, early oral or enteral feeding after safety evaluation, bedside limb and respiratory training, gradual ambulation, and daily assessments with timely adjustments to the care plan as needed.

All interventions were delivered by specialized nursing staff to ensure standardization and reproducibility.

### 2.3. Observation indicators

1. General information: Age, sex, body mass index, smoking and alcohol history, Charlson comorbidity index, American Society of Anesthesiologists classification, tumor location, clinical stage, and receipt of neoadjuvant therapy.
2. Surgical parameters: Surgical approach (minimally invasive or open), operative time, intraoperative blood loss, whether transfused, number of chest drainage tubes, and postoperative intensive care unit admission.
3. SUPPH scale: The Chinese version of the cancer self-management efficacy scale includes 28 items across 3 domains – self-decision-making (3 items), positive attitudes (15 items), and stress reduction (10 items) – rated on a 5-point Likert scale. The total score ranges from 28 to 140, categorized as low (28–55), medium (56–112), and high (113–140). Assessments were conducted on day 2 of admission, postoperative day 1, and prior to discharge.
4. ESCA scale: The Chinese version consists of 43 items, scored on a 5-point Likert scale with a total of 0 to 172 points. Higher scores reflect stronger self-care ability, categorized as low (<56), medium (56–114), and high (>114). Assessments were conducted on day 2 of admission, postoperative day 1, and before discharge.
5. MDASI (Chinese version): This instrument includes 13 symptom-severity items and 6 activity-interference items, each scored from 0 to 10. Severity was classified as mild (0–3), moderate (4–6), or severe (7–10); functional limitations were categorized similarly. Evaluations were performed on day 2 of admission, postoperative day 1, and prior to discharge.

### 2.4. Data collection and quality control

Patients completed the questionnaires under standardized instructions provided by trained researchers, and responses were reviewed and collected on site. All data were double-entered and independently verified by 2 investigators to ensure accuracy and reliability.

### 2.5. Ethics

This study was approved by the Ethics Committee of The First People’s Hospital of Kashi Prefecture, and written informed consent was obtained from all participants. The research adhered to the principles of voluntariness, informed participation, and confidentiality, and was conducted in accordance with the Declaration of Helsinki.

### 2.6. Statistical analysis

All analyses were 2-sided. Continuous variables were expressed as mean ± standard deviation (*x̄* ± *s*) and compared between groups using independent-samples *t* tests. Categorical variables were presented as counts and percentages, with group comparisons performed using the χ^2^ test or Fisher exact test when expected frequencies were < 5. For repeated measures across multiple time points and indicators, Bonferroni correction was applied to adjust for type I error. Statistical analyses were conducted using SPSS version 26.0 (IBM Corp., Armonk), with *P* < .05 considered statistically significant.

## 3. Results

### 3.1. Comparison of baseline characteristics

No significant differences were observed between the 2 groups with respect to age, sex, body mass index, smoking and alcohol history, Charlson comorbidity index, American Society of Anesthesiologists classification, tumor location, clinical stage, or receipt of neoadjuvant therapy (all *P* > .05), indicating comparability of baseline characteristics (Table 1).

**Table 1:** Baseline characteristics of patients in the ADOPT nursing group and the conventional care group.

Variable		The ADOPT nursing group (n = 38)	The conventional care group (n = 38)	*T*/χ^2^ value	*P* value
Age		62.5 ± 8.7	63.1 ± 9.1	−0.336	.737
Gender	Male	29 (76.3%)	29 (76.3%)	0.031	.861
	Female	9 (23.7%)	9 (23.7%)		
BMI		22.1 ± 2.8	21.8 ± 3.0	0.515	.607
Smoking	Yes	24 (63.2%)	24 (63.2%)	0.007	.935
	No	14 (36.8%)	14 (36.8%)		
Alcohol history	Yes	21 (55.3%)	21 (55.3%)	0.001	.974
	No	17 (44.7%)	17 (44.7%)		
Charlson comorbidity index		2.1 ± 1.2	2.0 ± 1.3	0.348	.729
ASA grading	Ⅰ	4 (10.5%)	3 (7.9%)	0.238	.971
	Ⅱ	17 (44.7%)	17 (44.7%)		
	Ⅲ	14 (36.8%)	15 (39.5%)		
	Ⅳ	3 (7.9%)	3 (7.9%)		
Tumor site	Upper	4 (10.5%)	4 (10.5%)	0.081	.960
	Middle	14 (36.8%)	15 (39.5%)		
	Lower	20 (52.6%)	19 (50.0%)		
Clinical stage	Ⅰ	6 (15.8%)	6 (15.8%)	0.067	.967
	Ⅱ	15 (39.5%)	16 (42.1%)		
	Ⅲ	17 (44.7%)	16 (42.1%)		
Neoadjuvant therapy	Yes	13 (34.2%)	12 (31.6%)	0.059	.808
	No	25 (65.8%)	26 (68.4%)		

ASA = American Society of Anesthesiologists, BMI = body mass index.

### 3.2. Comparison of surgical parameters

No significant differences were found between the 2 groups in surgical approach, operative time, intraoperative blood loss, whether transfused, number of chest drains, or postoperative intensive care unit admission (all *P* > .05) (Table 2).

**Table 2:** Surgical parameters of patients in the ADOPT nursing group and the conventional care group.

Variable		The ADOPT nursing group (n = 38)	The conventional care group (n = 38)	*T*/χ^2^ value	*P* value
Surgical approach	Luminal	29 (76.3%)	28 (73.7%)	0.070	.791
	Open	9 (23.7%)	10 (26.3%)		
Operative time		215.6 ± 48.3	218.9 ± 52.1	−0.328	.744
Intraoperative blood loss		180.4 ± 95.2	189.7 ± 109.8	−0.451	.653
Receipt of transfusion	Yes	3 (7.9%)	2 (5.3%)	0.214	.643
	No	35 (92.1%)	36 (94.7%)		
Number of chest drains	2	14 (36.8%)	11 (28.9%)	0.594	.743
	3	21 (55.3%)	23 (60.5%)		
	4	3 (7.9%)	4 (10.5%)		
Postoperative ICU admission	Yes	2 (5.3%)	1 (2.6%)	0.347	.556
	No	36 (94.7%)	37 (97.4%)		

ADOPT = Attitude, Definition, Open mind, Planning, Try it out, ICU = intensive care unit.

### 3.3. Comparison of SUPPH scale

On day 2 of admission and postoperative day 1, the distribution of SUPPH score levels was comparable between the 2 groups (*P* > .05). By discharge, however, the ADOPT group showed a significantly higher proportion of patients with high scores and a lower proportion with low scores, whereas changes in the conventional group were not significant. The between-group difference at discharge was statistically significant (*P* < .05) (Table 3).

**Table 3:** SUPPH score levels in the ADOPT nursing group and conventional care group at different assessment time points.

Variable	Day 2 of admission		Postoperative day 1		Before discharge	
	The ADOPT nursing group (n = 38)	The conventional care group (n = 38)	The ADOPT nursing group (n = 38)	The conventional care group (n = 38)	The ADOPT nursing group (n = 38)	The conventional care group (n = 38)
Low level (28–55)	15 (39.5%)	16 (42.1%)	14 (36.8%)	15 (39.5%)	5 (13.2%)	14 (36.8%)
Medium level (56–112)	18 (47.4%)	17 (44.7%)	19 (50.0%)	17 (44.7%)	13 (34.2%)	16 (42.1%)
High level (113–140)	5 (13.2%)	5 (13.2%)	5 (13.2%)	6 (15.8%)	20 (52.6%)	8 (21.1%)
χ^2^ value	0.061		0.237		9.716	
*P* value	.970		.888		.008	

ADOPT = Attitude, Definition, Open mind, Planning, Try it out, SUPPH = Strategies Used by People to Promote Health.

### 3.4. Comparison of ESCA scale

On day 2 of admission and postoperative day 1, no significant differences in ESCA score distribution were observed between the 2 groups (*P* > .05). During rehabilitation, however, the proportion of patients with high scores in the ADOPT group increased steadily, reaching 57.9% before discharge, compared with 21.1% in the conventional group. This difference was statistically significant (*P* < .05), indicating a greater improvement in self-care ability in the ADOPT group (Table 4).

**Table 4:** ESCA score levels in the ADOPT nursing group and conventional care group at different assessment time points.

Variable	Day 2 of admission		Postoperative day 1		Before discharge	
	The ADOPT nursing group (n = 38)	The conventional care group (n = 38)	The ADOPT nursing group (n = 38)	The conventional care group (n = 38)	The ADOPT nursing group (n = 38)	The conventional care group (n = 38)
Low level (<56)	14 (36.8%)	15 (39.5%)	12 (31.6%)	14 (36.8%)	4 (10.5%)	12 (31.6%)
Medium level (56–114)	20 (52.6%)	19 (50.0%)	21 (55.3%)	20 (52.6%)	12 (31.6%)	18 (47.4%)
High level (>114)	4 (10.5%)	4 (10.5%)	5 (13.2%)	4 (10.5%)	22 (57.9%)	8 (21.1%)
χ^2^ value	0.060		0.289		11.733	
*P* value	.970		.865		.003	

ADOPT = Attitude, Definition, Open mind, Planning, Try it out, ESCA = Exercise of Self-Care Agency.

### 3.5. Comparison of MDASI scale

On day 2 of admission and postoperative day 1, no significant differences were observed between the 2 groups in symptom severity or functional limitation (*P* > .05). By discharge, however, the ADOPT group showed a marked increase in the proportion of patients with mild symptoms and functional limitations, along with a significant reduction in severe cases, whereas improvements in the conventional group were minimal. The between-group differences at discharge were statistically significant (*P* < .05) (Tables 5 and 6).

**Table 5:** MDASI symptom severity in the ADOPT nursing group and conventional care group at different assessment time points.

Symptom severity	Day 2 of admission		Postoperative day 1		Before discharge	
	The ADOPT nursing group (n = 38)	The conventional care group (n = 38)	The ADOPT nursing group (n = 38)	The conventional care group (n = 38)	The ADOPT nursing group (n = 38)	The conventional care group (n = 38)
Mild (0–3)	10 (26.3%)	9 (23.7%)	14 (36.8%)	11 (28.9%)	24 (63.2%)	16 (42.1%)
Moderate (4–6)	18 (47.4%)	18 (47.4%)	18 (47.4%)	18 (47.4%)	11 (28.9%)	16 (42.1%)
Severe (7–10)	10 (26.3%)	11 (28.9%)	6 (15.8%)	9 (23.7%)	3 (7.9%)	6 (15.8%)
χ^2^ value	0.100		0.807		8.200	
*P* value	.951		.668		.017	

ADOPT = Attitude, Definition, Open mind, Planning, Try it out, MDASI = MD Anderson Symptom Inventory.

**Table 6:** MDASI functional limitations in life activities in the ADOPT nursing group and conventional care group at different assessment time points.

Functional limitations	Day 2 of admission		Postoperative day 1		Before discharge	
	The ADOPT nursing group (n = 38)	The conventional care group (n = 38)	The ADOPT nursing group (n = 38)	The conventional care group (n = 38)	The ADOPT nursing group (n = 38)	The conventional care group (n = 38)
Mild restriction (0–3)	11 (28.9%)	10 (26.3%)	14 (36.8%)	12 (31.6%)	25 (65.8%)	16 (42.1%)
Moderate restriction (4–6)	19 (50.0%)	19 (50.0%)	19 (50.0%)	20 (52.6%)	11 (28.9%)	16 (42.1%)
Severe restriction (7–10)	8 (21.1%)	9 (23.7%)	5 (13.2%)	6 (15.8%)	2 (5.3%)	6 (15.8%)
χ^2^ value	0.106		0.719		8.000	
*P* value	.948		.698		.018	

ADOPT = Attitude, Definition, Open mind, Planning, Try it out, MDASI = MD Anderson Symptom Inventory.

## 4. Discussion

### 4.1. Positive effect of the ADOPT nursing model on self-efficacy

This study demonstrated that, prior to discharge, the proportion of patients with high SUPPH scores was significantly greater in the ADOPT group than in the conventional group, indicating that the model effectively enhances self-efficacy. Self-efficacy is recognized as a key psychological determinant of treatment adherence and rehabilitation behaviors in cancer patients, with higher levels promoting initiative and motivation throughout treatment and recovery.^[19,20]^ The ADOPT model establishes a closed-loop management pathway through attitude guidance, goal definition, open-mindedness, individualized planning, and dynamic implementation. This framework not only builds patients confidence preoperatively but also provides continuous positive reinforcement postoperatively. Our findings are consistent with those of Lawler et al^[16]^ and Zhou et al,^[17]^ while further underscoring the systemic advantages of the ADOPT model in strengthening self-efficacy. Such benefits may derive from the model emphasis on personalized assessment, ongoing follow-up, and reinforcement of patients sense of control and engagement during care. Unlike prior studies that focused mainly on health promotion, the present study highlights the ADOPT model as a more continuous and actionable intervention pathway, thereby yielding greater improvements in self-management. The findings suggest that the ADOPT nursing model can improve short-term postoperative self-management efficacy, self-care ability, and symptom control among patients undergoing radical esophagectomy. However, long-term effects require further investigation.

### 4.2. Promotion of self-care ability by the ADOPT nursing model

According to the ESCA scale, 57.9% of patients in the ADOPT group reached a high self-care level before discharge, compared with 21.1% in the conventional group, indicating a significant advantage of the ADOPT model in improving self-care capacity. During the perioperative period, esophageal cancer patients often face nutritional deficiencies, physical decline, and psychosocial adaptation challenges, all of which can hinder recovery without effective self-care.^[21,22]^ Traditional nursing tends to rely on single health education measures, lacking continuity and individualization, which limits the development of sustainable self-care behaviors. In contrast, the ADOPT model incorporates individual differences and multidisciplinary input during the “definition” and “planning” stages, facilitating comprehensive care plans that include nutritional guidance, exercise training, pain management, and psychological support. Zhang et al^[23]^ demonstrated that structured health education improves self-care ability in patients with gastrointestinal tumors. The present findings further support this conclusion, showing that the ADOPT model not only strengthens physiological care behaviors but also enhances psychological and social functioning through closed-loop management. Moreover, a meta-analysis by Qin et al^[24]^ confirmed that psychological interventions significantly improve coping styles and quality of life. In this study, the psychological care and open communication mechanisms embedded in the ADOPT model appear to have played a central role in the marked improvement in self-care ability.

### 4.3. Combined improvement in symptom clusters and functional recovery with the ADOPT model

MDASI results indicated that patients in the ADOPT group experienced significant reductions in symptom severity and functional limitations before discharge, whereas improvements in the conventional group were limited. These findings suggest that the ADOPT model extends beyond single-symptom management, offering advantages in comprehensive symptom cluster control and functional recovery. Postoperatively, esophageal cancer patients frequently suffer from pain, fatigue, sleep disturbances, and anxiety, which often occur concurrently and interact synergistically, worsening discomfort and impairing quality of life. In this study, the ADOPT model addressed these challenges through multifaceted measures, including preoperative functional exercises, early postoperative mobility guidance, integrated pain management, and nutritional support. This dual focus on physiological and psychological needs facilitated multidimensional symptom relief. In line with findings from Chen et al^[8]^ and Cui et al,^[6]^ our results reinforce that single-symptom strategies are inadequate for complex perioperative demands, whereas systematic interventions achieve broader improvements through synergistic effects. The demonstrated strength of this model in symptom cluster management highlights the need for future esophageal cancer care to shift from isolated interventions toward multidimensional, integrated approaches.

### 4.4. Clinical significance and promotion value

Clinically, the ADOPT model enhances self-efficacy, self-care ability, and symptom cluster control in the short term, while establishing a foundation for long-term recovery. By providing systematic and individualized interventions, it supports sustained self-management behaviors after discharge, thereby reducing recurrence risk and improving quality of life. Importantly, this model complements the existing enhanced recovery after surgery (ERAS) pathway: whereas ERAS emphasizes optimization of physical aspects of perioperative care, the ADOPT model strengthens psychological support, patient engagement, and multidisciplinary integration. The combination of the 2 may further advance systematic and standardized perioperative management for esophageal cancer.^[25,26]^ Moreover, the applicability of the ADOPT model extends beyond esophageal cancer to other gastrointestinal tumors such as gastric and colorectal cancer. It also holds potential value in chronic disease management and multimorbidity care, and could become an integral component of broader clinical care pathways.

### 4.5. Limitations and future prospects

Although this study yielded positive results, several limitations should be acknowledged. First, it was a single-center trial with a relatively small sample size, which may limit external generalizability. Future multi-center, large-sample randomized controlled trials are needed to validate and extend these findings. Second, the follow-up was restricted to the hospitalization period, making it insufficient to evaluate the long-term effects of the ADOPT model on quality of life and survival. Third, although internationally validated scales were employed, they may not fully capture esophageal cancer–specific issues such as dysphagia and nutrition-related complications; thus, incorporating localized, disease-specific instruments for the Chinese population is warranted. Finally, potential confounders such as psychological status and social support were not fully controlled, which may have influenced the observed intervention effects. Because participants and nursing staff could not be blinded to group allocation, performance bias cannot be completely excluded, which should be considered when interpreting the findings.

Future research may proceed in several directions. First, larger multi-center trials with extended follow-up are needed to clarify the long-term impact of the ADOPT model on recovery and survival. Second, strengthening interdisciplinary collaboration to develop esophageal cancer–specific nursing assessment tools will improve the precision and individualization of interventions. Third, integrating the ADOPT model with digital health technologies – such as mHealth applications, telemedicine, and instant messaging platforms – could enable extended, intelligent, and more accessible nursing interventions.^[27]^ Advancing these areas will further enhance the effectiveness of nursing care, improve patient adherence, and facilitate the broader clinical adoption of the ADOPT model. While the ADOPT nursing model demonstrated promising short-term benefits, future longitudinal studies are needed to evaluate sustained outcomes. Integration of ADOPT principles with ERAS pathways may represent a valuable direction for future research.

## Author contributions

**Conceptualization:** Pazilaiti Saideng, Yuan Lv, Gulizhaernuer Aierken, Mayire Tuersun, Minawaer Wujiaji.

**Data curation:** Pazilaiti Saideng, Yuan Lv, Gulizhaernuer Aierken, Mayire Tuersun, Minawaer Wujiaji.

**Formal analysis:** Pazilaiti Saideng, Yuan Lv, Gulizhaernuer Aierken, Mayire Tuersun, Minawaer Wujiaji.

**Funding acquisition:** Pazilaiti Saideng, Gulizhaernuer Aierken, Mayire Tuersun, Minawaer Wujiaji.

**Investigation:** Minawaer Wujiaji.

**Writing – original draft:** Pazilaiti Saideng, Yuan Lv, Minawaer Wujiaji.

**Writing – review & editing:** Pazilaiti Saideng, Yuan Lv, Minawaer Wujiaji.
